# The impact of COVID-19 on inflammatory bowel disease surgery: a systematic review

**DOI:** 10.1308/rcsann.2025.0016

**Published:** 2025-04-24

**Authors:** J Couch, C Li, K Thomas, T Card, D Humes

**Affiliations:** Nottingham University Hospitals NHS Trust, UK

**Keywords:** COVID-19, Pandemics, Inflammatory bowel diseases, Ulcerative colitis, Crohn’s disease, Surgical procedures

## Abstract

**Introduction:**

The COVID-19 pandemic caused a significant disruption to the delivery of surgical services. Guidance prioritising life-saving and cancer surgery was issued. Inflammatory bowel disease (IBD) often requires considered, timely surgery, which may have not been feasible under the conditions imposed by the pandemic. This systematic review aims to quantify the impact of COVID-19 on IBD surgery and assess the safety of performing such surgery.

**Methods:**

A systematic review of MEDLINE, Embase and Web of Science was performed. Studies that included a prepandemic and a pandemic cohort for comparison and reported on numbers of IBD surgeries or postoperative outcomes following IBD surgery were included. Heterogeneity of included studies precluded any meta-analyses.

**Findings:**

In total, 1,220 titles were screened and 13 were included in the final review. All were cohort studies other than one case–control study. A total of 1,673,282 and 1,445,971 patients were included in the prepandemic and pandemic cohorts, respectively. Rates of elective surgery during the pandemic varied from a 66% reduction to a 9.66% increase and emergency surgery varied from no difference to an 18% reduction. Urgent surgery in IBD inpatients appears to be unaffected. Postoperative outcomes were not shown to be negatively impacted by resource limitations.

**Conclusions:**

The COVID-19 pandemic affected IBD surgical services considerably; however, those who did undergo surgery during this period do not appear to have been at an increased risk of adverse outcomes. Further work is required to describe the long-term impacts of these cancellations on IBD services and patient morbidity.

## Introduction

In March 2020, the World Health Organization declared the coronavirus (COVID-19) outbreak a global pandemic.^1^ This led to an unprecedented overhaul of healthcare delivery and a significant reduction in utilisation.^2^ Inflammatory bowel disease (IBD) is primarily managed with medication; however, it has been estimated that 16% of Crohn’s disease (CD) patients and 5% of patients with ulcerative colitis (UC) will require surgery in the first year following diagnosis.^3^ Owing to the chronic, relapsing and remitting nature of IBD, patients benefit from regular access to outpatient follow-up to ensure disease-controlling surgery can be delivered in a timely manner. Delays in IBD diagnoses and surgery have been shown to increase the need for surgery and postoperative complications.^4,5^ This can have negative implications on morbidity, quality of life and psychological wellbeing, and can result in substantial financial costs to both the economy and individual.^6,7^

At the start of the pandemic, there was an almost complete cessation of non-urgent care to preserve resources for COVID-19-related admissions.^2^ Colorectal surgery was no exception, and guidelines were issued recommending cancer care be prioritised over benign disease.^8^ Specific guidance was issued advising the deferral of non-life-saving IBD surgeries with an acceptance of fewer laparoscopic surgeries and higher stoma rates.^9,^^10^ The impact of this pandemic-induced strain on IBD service delivery is unclear. We performed a systematic review to determine how COVID-19 affected the number of IBD-related surgeries and postoperative outcomes to see whether healthcare systems were able to mitigate the initial effects of the pandemic. This may provide some guidance on how IBD surgical services need to recover and might be safe-guarded in times of future resource scarcity.

## Methods

We conducted a systematic review using a predefined protocol that was registered with the International Prospective Register of Systematic Reviews (PROSPERO; CRD42023427363), in accordance with the Preferred Reporting Items for Systematic Reviews and Meta-Analyses (PRISMA) statement.^11^

### Search strategy

A search of electronic databases MEDLINE, Embase and Web of Science was performed on 15 July 2024. The search strategy used for PubMed (Appendix 1 – available online) was developed with the assistance of a research librarian from the University of Nottingham. The strategy was adapted to each database. Date restrictions from 1 January 2020 to the 15 July 2024 were used. Search results were uploaded to Rayyan for screening of titles and abstracts by two independent reviewers (JC and DL).^12^ Conflicts were resolved following discussion between the two reviewers until a consensus had been reached. Full-text articles were reviewed by the corresponding author (JC).

### Selection criteria

Studies that compared prepandemic and pandemic numbers of IBD-related surgeries (primary outcome) or outcomes of IBD-related surgeries (secondary outcome) were included. Studies were excluded if they did not have a prepandemic historical cohort for comparison, included paediatric patients or if cohorts contained fewer than ten patients. Surveys, audits, editorials, letters to the editor, reviews, guidelines or opinions were excluded. There were no language or geographic restrictions.

### Data extraction and analysis

Data were extracted by reviewer JC. For each included study, data were collected for the study design, country of origin, periods that defined the prepandemic and pandemic cohorts, number of participants, IBD-related surgeries, whether surgeries were defined as elective or emergency, the use of laparoscopy, postoperative complications, length of stay and reoperation.

### Assessment of study quality and risk of bias

None of the studies included were randomised controlled trials and therefore the Newcastle–Ottawa Scale (NOS) was used to assess for quality and bias.^13^ The NOS assesses studies against selection criteria (representativeness of cohorts and ascertainment of exposure and outcome), comparability (whether the study controls for confounders) and outcome (how it was assessed, and length and completeness of follow-up). A total score out of 9 can be given with scores of 7 or more being considered as higher quality and those 3 or less as lower quality.

### Data synthesis and analysis

For studies comparing the number of IBD surgeries in the prepandemic and pandemic periods, heterogeneity in reporting between studies precluded a meta-analysis of the primary outcome. Similarly, for studies reporting postoperative outcomes, there was clinical heterogeneity in the type of surgery performed and admission modality, again precluding a meaningful meta-analysis.

## Findings

### Studies identified

**Figure 1:**
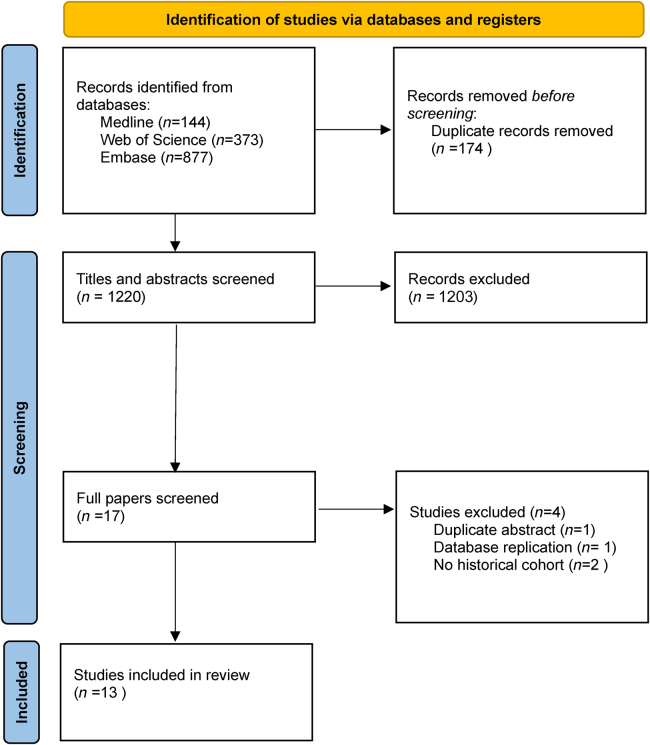
PRISMA diagram

A total of 1,621 studies were identified from database searches; 401 duplicates were removed leaving 1,220 titles and abstracts for screening by reviewers. Of these, 1,203 were excluded for not meeting inclusion criteria based on title or abstract. Of the 17 papers that went on to full screening, 4 were excluded: 1 because it was a conference abstract for a published paper already included; 1 because it used the same database, albeit with a shorter follow-up time for a paper already included; and 2 because they did not include a historical cohort (Figure 1). Two studies from the Netherlands, three from England and three studies from the USA may include overlapping patient cohorts owing to single-centre study patients being included in national databases or patients being included in multiple national databases.^4,14–20^ Considering that a meta-analysis was not performed, all of these studies were included to maximise comprehensiveness because the aforementioned studies report different outcome measures and allowed for comparison of practice at both a national and local level.

### Quality of studies

Using the NOS for assessment, four studies were deemed high quality, seven were of moderate quality and two were considered to be low quality.^4,14–25^ Most studies scored well in selection criteria domains, but few studies met the criteria for ensuring comparability of cohorts and adequacy of follow-up (Appendix 1 – available online).

### Study characteristics

The systematic review encompassed 3,125,253 patients in total; 1,673,282 in the prepandemic cohort and 1,445,971 in the pandemic cohort. It is possible that 4,241 patients in the prepandemic cohort and 3,879 in the pandemic cohort were represented twice because of the overlapping of studies. Of the 13 studies included, 11 were retrospective cohort studies, 1 was a cross-sectional study and 1 was a case–control study. Four studies were from the USA, three from England, two from the Netherlands, two from Italy and one was from Canada. Five of the studies utilised routinely collected data from national databases. Deputy *et al* used Hospital Episode Statistics (HES), which collects data from all hospital admissions funded by the National Health Service; Derks *et al* used the Automated National Pathological Anatomy Archive (PALGA), which receives histological specimens from every pathology laboratory in the Netherlands; Ungaro *et al* drew data from Symphony Health Integrated Dataverse, which covers 280 million people’s health insurances claims in the USA; Nanah *et al* used Nationwide Inpatient Sampling, which the largest, publicly available inpatient care database in the USA which encompasses all payer types; and Ghodasara *et al* used the National Surgical Quality Improvement Project (NSQIP), which collects data from surgical outcomes from more than 800 hospitals in the USA.^14,16,19,20,22^ The defined pandemic period for all studies other than two did not extend beyond 2020 (Table 1).

**Table 1:** Characteristics of studies included in systematic review

Author (year)	Study design	Country	Prepandemic/pandemic period	No. of patients in cohort (prepandemic/pandemic)	Outcomes of interest measured	Summary of outcomes (prepandemic vs pandemic)	Newcastle–Ottawa score
Strobel *et al* (2024)^21^	Retrospective cohort, single centre	Germany	1 February 2015 to 25 May 2018/1 March 2020 to 15 December 2020	107/97	Surgical site infections in elective IBD resections	31.8% vs 25.8% (*p *= **0.345**)	6
					Postoperative complications	19.6% vs 24.7% (*p *= 0.181)	
					Reoperation	18.7% vs 15.5% (*p *= 0.541)	
					Length of stay	11 days vs 8 days (*p *<** 0.001**)	
De Bock *et al* (2023)^4^	Retrospective cohort, multicentre	Netherlands	16 March 2020 to 31 December 2020/16 March 2019 to 31 December 2019	46/35	Number of elective and emergency IBD surgeries	Elective: 38 (82.6%) vs 25 (71.4%) Emergency: 8 (17.4%) vs 10 (28.6%) (*p *= 0.352)	7
					Laparoscopic approach	24 (52.2%) vs 20 (57.1%) (*p *= 0.966)	
					Postoperative complications	8 (17.4%) vs 11 (31.4%) (*p *= 0.225)	
Derks *et al* (2024)^14^	Retrospective cohort using national database (PALGA)	Netherlands	March 2018 to February 2020/March 2020 to February 2022	2,722/2,740	Number of IBD-related surgeries	+0.7% increase in pandemic period	4
Yeung *et al* (2022) ^15^	Retrospective cohort, single centre	England	1 March to 31 June 2019/1 March 2020 to 31 June 2020	50/17	Number of elective IBD resections	50 vs 17 (66% reduction)	4
Ungaro *et al* (2022)^22^	Cross-sectional using national database (Symphony Health Integrated Dataverse)	USA	2019/2020	1,318,414/1,292,459	IBD-related surgeries	3.14 per 1,000 patients with IBD per month vs 2.80 per 1,000 patients with IBD per month	4
Pastena *et al* (2022)^23^	Retrospective cohort, single centre	Italy	2019/2020	77/83	Total number of IBD surgeries	77/83 (4.6% increase)	5
					Length of stay	7 days vs 6 days	
					Laparoscopic approach	49% vs 59%	
					Postoperative complications	6.5% vs 4.8%	
Deputy *et al* (2022)^16^	Observational, using national database (Hospital Episode Statistics)	England	1 January 2015 to 31 January 2020/1 February 2020 to 31 January 2021	166,948/32,107	Predicted deficit of IBD surgeries during pandemic period	Elective colectomy: 18%	7
						Emergency colectomy: 12.1%	
						CD resection/strictutreplasty: 12.7%	
						CD fistula surgery: 36.9%	
						Ileostomy reversal: 40.8%	
Sebastian *et al* (2021)^17^	Case–control, multicentre	England	1 January 2019 to 20 June 2019/1 March 2020 to 30 June 2020	398/384	Number of emergency colectomies in acute severe ulcerative colitis admissions	13% vs 16% (*p *= 0.26)	7
					Laparoscopic approach	76% vs 53% (*p *= **0.018**)	
Odufalu *et al* (2024)^18^	Observational, multicentre	USA	March 2019 to December 2019/March 2020 to December 2020	75/52	Number of IBD-related surgeries	75 vs 52 (*p *= 0.72)	4
Malhi *et al* (2022) ^24^	Retrospective cohort study, multicentre	Canada	17 March 2019 to 31 August 2019/17 March 2020 to 31 August 2020	709 vs 520	Number of inpatient surgeries	22.7% vs 15.6% (*p *= 0.19)	3
Nardone *et al* (2020) ^25^	Retrospective observational	Italy	January to February 2020/March to April 2020	881/971	Number of surgeries	10 vs 9 (*p *= 0.82)	3
Nanah *et al* (2023)^19^	Retrospective cohort, using national database (nationwide inpatient sampling)	USA	January 2017 to March 2019/April 2019 to December 2020	179,119/113,052	Number of emergency colectomies	UC: 2.9% vs 3.25% (*p *= 0.253) CD: 0.92% vs 0.89% (*p *= 0.745)	7
					Length of stay	UC: 5.03 vs 5.2 CD: 4.67 vs 4.65	
					Need for organ support	Mechanical ventilation: UC 2.6% vs 5.5% (*p *= **0.0004**) CD 0.13% vs 0.26% (*p *= 0**.0043**) Vasopressors: UC: 1.9% vs 2.8% (*p *= 0.178) CD: 0.16% vs 0.25% (*p *= 0.52)	
Ghodasara *et al* (2024)^20^	Retrospective cohort using national database (National Surgical Quality Improvement Program)	USA	2019 vs 2020	3,747/3,443	Number of IBD colectomies	3,747 vs 3,443	5
					Postoperative complications	11.90% vs 12.11%	
					Reoperation	5.18% vs 5.63%	

CD = Crohn’s disease; IBD = inflammatory bowel disease; PALGA = Automated National Pathological Anatomy Archive; UC = ulcerative colitis

### Elective resections

Three studies reported on the change in the number of elective colectomies.^15,16,20^ Yeung *et al* reported that the number of colectomies for IBD reduced from 50 to 17 (66% reduction) during the pandemic period compared with the same period of the preceding year.^15^ Deputy *et al* found there to be an 18% deficit in elective colectomies when using autoregressive integrated moving average forecast models to predict the anticipated number of surgeries during the pandemic compared with the numbers recorded in a national database of routinely recorded hospital admissions (HES).^16^ Ghodasara *et al* using data from NSQIP reported that the proportion of elective colectomies increased for both UC (476/1,056 [45.08%] vs 556/980 [56.73%]) and CD (1,975/2,690 [73.42%] vs 1,996/2,463 [81.04%]) from 2019 to 2020, respectively.^20^

### Emergency resections

Four studies compared the number of emergency colectomies performed during the pandemic period with a historical cohort.^16,17,19^ Sebastian *et al* performed a multicentre, case–control study comparing patients who presented to secondary care with severe acute UC and found colectomy rates were similar between the pandemic and control group (64/389 [16%] vs 50/375 [13%], *p *= 0.26).^17^ Nanah *et al* also found similar number of colectomies among emergency IBD admissions for both UC (2.9% [1,995/68,739] vs 3.25% [1,635/50,307], *p *= 0.25) and CD (0.92% [1,015/110,326] vs 0.89% [705/79,213], *p *= 0.74).^19^ Deputy *et al* reported an 12.1% deficit based on predictive forecast models, whereas Ghodasara *et al* reported the proportion of colectomies performed as an emergency procedure decreased for both UC (581/1,057 [54.97%] vs 242/980 [24.69%], *p *= 0.000) and CD (715/2,690 [26.58%] vs 467/2,463 [18.96%], *p *= 0.000) from 2019 to 2020.^16,^^20^

### Unspecified resections

One study did not specify the admission type of the colectomies reported. De Bock *et al* reported colectomy-specific data in their supplementary material demonstrating that a similar number of colectomies were performed between March and December 2020 and the same period of the preceding year (27 vs 28).^4^

### IBD-related surgeries

Six studies encompassed all IBD-related surgeries.^4,16,22–25^. De Bock *et al* and Pastena *et al* both reported an increase, 10 vs 17 (non-colectomy IBD surgeries) and 4.6% respectively, and Malhi *et al* reported a 50% increase in inpatient surgeries (*p *= 0.39).^4,23,24^ Nardone *et al* reported similar numbers of surgeries (10/881 vs 9/971, *p*=0.82) when comparing pandemic and prepandemic cohorts.^25^ Two studies reported a reduction in IBD-related surgeries.^16,22^ Ungaro *et al*, using the NSQIP database, reported a reduction from 3.14 per 1,000 IBD surgeries in 2019 to 2.88 per 1,000 IBD surgeries in 2020 and Deputy *et al* estimated deficits of 12.7% for CD resections, 40.8% for ileostomy reversal and 36.9% for CD fistula surgery.^16,22^

### Major complications

Four studies reported major complications following IBD surgery, defined as a Clavien–Dindo classification grade of 3 or more.^4,20,21,23,^^25^ Strobel *et al* and de Bock *et al* both reported a non-significant increase in postoperative complications during the COVID-19 pandemic of 9.6% vs 24.7% (*p *= 0.181)^21^ and 17.4% vs 31.4% (*p *= 0.255), respectively. Pastena *et al* and Ghodasara *et al* reported a similar number of complications comparing the periods of 6.5% vs 4.8%, and 11.90% (446/3,747) vs 12.11% (417/3,443), respectively.^4,23^

Strobel *et al* specifically reported number of surgical site infections (SSI). No statistically significant difference was found in the number of SSI following elective IBD resections (31.8% vs 25.8%, *p *= 0.345); however, when reviewing postoperative intra-abdominal abscesses, there were significantly more during the pandemic period (7.2% vs 0.9%, *p *= 0.021).^21^

### Secondary outcomes

#### Minimally invasive surgery

Four studies reported the proportion of minimally invasive surgery performed for resectional surgery. Three studies reported an increase in the use of laparoscopic surgery.^4,21,23^ Strobel *et al*, who reported elective resections, found only a small increase (51.5% vs 55.7%, *p *= 0.479).^21^ De Bock *et al* and Pastena *et al* included both elective and emergency resections and also reported a similar small non-significant increase in laparoscopic surgery (52.2% vs 57.1% [*p *= 0.99] and 49% vs 59%, respectively).^4,23^ The study by Sebastian *et al* looking specifically at acute severe UC colectomies found a statistically significant reduction in laparoscopic surgery rates (38/50 [76%] vs 34/64 [53%], *p *= 0.018).^17^

#### Other reported outcomes

Three studies reported postoperative length of stay.^19,21,23^ Strobel *et al* reported a 3-day reduction in elective colectomy postoperative stays (11 vs 8, *p *< 0.001).^21^ Pastena *et al* reported a reduction from 7 to 6 days and Nanah *et al*, who looked exclusively at emergency admissions, found no difference.^19,^^23^

Ghodasara *et al* reported the number of readmissions following colectomy and found no difference when comparing the prepandemic and pandemic cohorts (194/3,747 [5.18%] vs 194/3,443 [5.63%]).^20^

Nanah *et al* found UC patients were more likely to be admitted with a perforation (1% vs 1.3%, *p *= 0.025) and both UC and CD patients were more likely to require mechanical ventilation (UC 2.6% vs 5.5%, *p *= 0.0004; CD 0.13% vs 0.26%, *p *= 0.0043). There was also an increase in vasopressor requirements for both CD and UC during the pandemic period although this did not reach statistical significance (UC 1.9% vs 2.8%, *p *= 0.178; CD 0.16% vs 0.25%, *p *= 0.052). Sebastian *et al*, however, found there to be no difference between intensive care admissions and need for ventilatory support.

Only one study, Nanah *et al*, reported the number of patients with concomitant COVID-19 infection at the time of surgery: 0.54% for UC and 0.40% for CD patients.^19^

## Discussion

This systematic review aimed to give an overview of the impact the COVID-19 pandemic had on IBD surgery in terms of volume and postoperative complications. As might be expected following guidance issued at the beginning of the pandemic period, the majority of studies reported that non-urgent IBD surgery reduced considerably. Ghodasara *et al* reported that the proportion of emergency colectomies decreased. This is surprising given what is understood about health-seeking behaviours during the pandemic, particularly in response to advice around shielding. In April 2020, the British Society of Gastroenterology (BSG) issued guidance advising high-risk patients to shield.^31^ It was not until May 2022 that this guidance was withdrawn, noting that most IBD patients were not at increased risk of adverse outcomes following COVID-19.^32^ It is likely that advice around shielding led to patients being reluctant to seek medical input for fear of contracting the virus. This and the redistribution of resources are likely to be the main factors contributing to the overall reduction in IBD surgery, particularly planned, non-urgent surgery. However, it is counterintuitive that the proportion of emergency surgeries would decrease because the majority of IBD patients undergoing surgery during the pandemic would be those with disease severity deeming urgent surgery unavoidable.

We must also consider how changes to medical management during the pandemic might have influenced the requirement or patient preference to undergo surgery. BSG guidelines at the start of the pandemic advised patients to continue immunomodulators and biologics on the basis of no known risk to doing so.^31^ However, the paucity of evidence supporting their safety may have made prescribers hesitant to do so. Evidence in the literature suggests that prescribing practices did change, with a reduction in the number of people starting an immunomodulator, a reduction in *de novo* prescriptions of infliximab and an increase in the use of biologics as monotherapy (as opposed to the concomitant use of an immunomodulator).^33,34^ Although patients might have had similar safety concerns, adherence to prescribed medications was high.^35–37^ Evidence suggests that patients were fearful of attending hospital and preferred care to be delivered remotely and, therefore, it seems unlikely that many patients would have opted for surgical treatments over medical therapies.^36,37^ In addition, early in the pandemic period evidence emerged reporting high mortality rates when contracting covid in the perioperative period, leading to higher thresholds for consideration of surgery.^38^ Studies demonstrating minimal impact of the pandemic on operative volume may be testament to the success of telemedicine, which had widespread implementation during the pandemic, a change in approach to IBD management with a preference for anti-inflammatory medications and interventional radiology, or the ability of some healthcare settings to utilise ‘COVID-19-free’ sites that were ring-fenced facilities enabling the majority of planned surgeries to go ahead as normal.^39^

Deputy *et al* report a deficit in the number of emergency colectomies (12.1%), which is in keeping with a reduction in overall attendances to secondary care for emergency IBD care. Reassuringly 30-day mortality and readmission rates for medical patients were similar to those prior to the pandemic, which suggests that those who required admission for IBD were managed appropriately. What remains unknown is the morbidity resulting from these apparently untreated emergency IBD cases or whether these patients have historically been over treated. There is also the possibility that a reduction in IBD-related hospitalisations and the avoidance of nosocomial infections, such as *Clostridium difficile*, resulted in less-acute severe flares during the pandemic.^40,41^ Both studies looking at emergency colectomies in patients who had already presented to acute care found colectomy numbers during the pandemic to be similar. This could be a self-selecting cohort by which the thresholds for admission were such that surgical treatment was inevitable. Sebastian *et al* found that during the COVID-19 pandemic a greater proportion of patients who presented with acute severe UC required rescue therapy, but this was primarily driven by an increase in the use of biologicals, ciclosporin and tofacitinib, and may be reflective of a change in practice during the pandemic period, but raises the question as to long-term implications of postponing emergency surgery.

None of the studies reporting on postoperative complications found a significant increase in the number of major complications (Clavien–Dindo score 3–5). This would suggest that although resources were stretched, standards of care were maintained and there was no increased risk to performing surgery in those in whom it was deemed necessary. Two studies found a reduced length of stay following IBD surgery, and Deputy *et al* also reported a reduced length of stay for all acute IBD admissions. This may be a consequence of increased senior input leading to more discharge decisions and an enthusiasm from patients to be discharged home to what was perceived as safer environment. Interestingly, only one study from England reported a reduction in the use of laparoscopy despite initial concerns that this was an aerosol-generating procedure and may perpetuate the spread of infection.^17^ Variation in outcomes reported by studies may be a reflection of guidance issued at a national level. For example, in the UK, Intercollegiate General Surgery guidance issued at the start of the pandemic advised against the routine use of laparoscopic surgery, whereas the Society of American Gastrointestinal and Endoscopic Surgeons (SAGES) issued less-restrictive guidance, advising on the use of filtration devices and advocating for the benefits of laparoscopic surgery in terms of complications and length of stay.^42,^^43^

The strengths of this systematic review are that it included five studies from large, national routinely collated databases, which gave views of service provision on a national level and may be less subject to sampling bias. Included studies were all from high-income countries, the majority of which have a predominantly public healthcare system, making the results generalisable. We also included conference abstracts in our study to reduce the risk of publication bias and increase comprehensiveness.

### Study limitations

One of the main weaknesses of this systematic review is that all but one study included pandemic data during 2020 only, and therefore it is difficult to ascertain whether healthcare systems were able to mitigate the initial effects of the pandemic or whether the long-term consequences of resource limitation in subsequent waves persisted. The results of included studies were quite heterogeneous, with papers showing both an increase and decrease in IBD surgery, and although it is interesting to note that there were variations in practice, it is difficult to ascertain why this might be the case. It may be because resources were only limited for a short time during particular waves of the pandemic, which hit countries at different times, and this level of detail is not reflected in the data, or that some centres had access to ‘hot’ and ‘cold’ sites or could utilise private sector hospitals and therefore were able to continue with a greater proportion of elective care. Another limitation of our study is the possibility of the overlapping patient cohorts introducing a potential source of bias. Patients from single-centre studies in England and the Netherlands may also be included in national databases, and similarly in the US there may be some overlap between the NSQIP and NIS databases. However, the inclusion of all eligible studies maximised the comprehensiveness of our review, optimising representation of patients and hospitals and reducing sampling bias that may be introduced by relying heavily on a potentially skewed data set. Studies also reported slightly different outcomes and differed in their data collection methods. This gave a broader understanding of surgical practice and highlighted differences in the practice at a local and national level. In the absence of performing a meta-analysis, we believe that the advantages of providing a more comprehensive overview of IBD surgery during the pandemic outweighs the limitations of possible duplication.

Considering the impact of COVID-19 on other types of colorectal surgery, a large population-based study from England looking at colorectal cancer surgery found that although cancer surgery had been prioritised, at the start of the pandemic there was a 31% reduction in colorectal cancer surgeries.^44^ This reduction in colorectal cancer surgery is echoed in studies from other countries such as Germany, Portugal and Australia, where resections reduced by up to 60%.^45–47^ Similar to the work included in our review, the majority of studies looking at colorectal cancer surgery found that there was no significant reduction in the use of laparoscopy, no increase in postoperative complications or need for intensive care, and reduced length of stay following surgery.^48^ Several studies that reviewed the presentation of colorectal cancer found that patients were more likely to present with symptomatic and advanced disease during the pandemic, as was the case in our review.^49–51^ It is also apparent that colorectal cancer surgery saw similar trends to that seen in the papers included in our review, whereby surgery numbers fluctuated throughout the first year of the pandemic, likely in response to repeated waves of COVID-19, when resources became particularly strained followed by a period of recovery.^22,52^

## Conclusions

The COVID-19 pandemic posed major challenges to the delivery of IBD services. Although elective surgery was heavily impacted, those unwell enough to require hospital admission were able to be operated on without increased risk. The implementation of telemedicine and improved efficiency with regards to postoperative discharges during the pandemic could serve to optimise resources in the post-pandemic era. Further work is required, first to describe the effects of the pandemic beyond 2020 and ascertain whether IBD services were able to sustain a recovery period sufficient enough to mitigate the impact of the first year of the pandemic; and second, to ascertain the long- term impact on patients whose surgery was postponed.
